# Maximizing horizontal transmission through mating: increased mating frequency and mating competitiveness associated with *Microsporidia MB*-infected *Anopheles arabiensis* males

**DOI:** 10.1186/s12936-025-05354-1

**Published:** 2025-04-09

**Authors:** Tracy Maina, Aclaine Shisia, Joseph Gichuhi, Joel L. Bargul, Jeremy K. Herren, Tullu Bukhari

**Affiliations:** 1 Global Health Thematic Research Programme, International Centre of Insect Physiology and Ecology (Icipe), P.O. Box 30772 - 00100, Nairobi, Kenya; 2 Department of Biochemistry, Jomo Kenyatta University of Agriculture and Technology (JKUAT), P.O. Box, 62000 - 00200, Nairobi, Kenya

## Abstract

**Background:**

*Microsporidia MB* is a naturally occurring symbiont in *Anopheles arabiensis* mosquitoes that inhibits the development of *Plasmodium.* It is transmitted both vertically and horizontally, enabling its spread within mosquito populations. Currently, mating is the only known mechanism for horizontal transmission. Understanding the factors that influence *Microsporidia MB* transmission during mating is crucial for developing a malaria transmission-blocking strategy based on this symbiont.

**Methods:**

The effect of mosquito age on *Microsporidia MB* transmission was determined through mating bioassays between infected and uninfected *Anopheles arabiensis* males and females in three age groups: 3–4 days, 7–8 days, and 10–11 days. Mating bioassays were also conducted to determine if *Microsporidia MB* infection affects the individual male mating frequencies and mating competitiveness of male mosquitoes. To assess the effect of *Microsporidia MB*-infection on swarming under field conditions, swarming and non-swarming *An. arabiensis* male mosquitoes were collected and compared for *Microsporidia MB* prevalence.

**Results:**

The age of mosquitoes does not affect the transmission of *Microsporidia MB* from males to females (χ^2^ = 11.6, df = 12, *p* = 0.47). However, transmission of the *Microsporidia MB* from female mosquitoes to males was not observed in the 3–4 days old age group. Although heterogeneous, there is higher overall transmission from male to female (41.5%) compared to female to male (22.4%). When individual males (*Microsporidia MB* infected or uninfected) were mated with females, *Microsporidia MB*-infected males on average mated two times more than the *Microsporidia MB*-uninfected F1 male mates from the age of 3–4 days to death (t = 2.2, df = 56.8, *p* = 0.03). Also, *Microsporidia MB*-infected males when combined in a cage with *Microsporidia MB* uninfected males were twice as competitive (χ^2^ = 4.74, df = 1, *p* = 0.02) to the uninfected males in mating with uninfected females. In natural swarms, the proportion of *Microsporidia MB*-infected males was significantly higher compared to the non-swarming male mosquitoes (χ^2^ = 685.5, df = 1, *p* < 0.0001).

**Conclusion:**

There is a moderate, although heterogenous, horizontal transmission of *Microsporidia MB* across all age groups, except from 3–4 days old, infected females to males. *Microsporidia MB*-infected male mosquitoes were almost twice as competitive in mating as their uninfected counterparts. Therefore, *Microsporidia MB* infected males can potentially disseminate *Microsporidia MB* in the natural mosquito populations, thus, contributing to malaria control. However, semi-field studies are required to validate these results in a natural environment.

**Supplementary Information:**

The online version contains supplementary material available at 10.1186/s12936-025-05354-1.

## Background

Malaria-endemic countries reported an estimated 249 million cases of malaria globally in 2022 [1]. This figure is 55% higher than the target level set by the 2025 Global Technical Strategy for malaria [2, 3]. While there is ongoing discussion about the accuracy of these estimates, it is widely acknowledged that the progress in malaria control has stalled [4, 5]. Factors like climate change and the spread of *Anopheles stephensi* in Africa are expected to exacerbate the situation, compounding existing challenges such as drug and insecticide resistance [1, 6, 7]. Consequently, there is an urgent need to enhance the current strategies and develop new approaches to control malaria [8].

*Microsporidia MB*, a natural symbiont of *Anopheles* mosquitoes, has been shown to block *Plasmodium* transmission without affecting the fitness of the mosquito host [9–12]. *Microsporidia MB* spreads both vertically—from mother to offspring—and horizontally through mating, enabling it to propagate throughout the mosquito populations [9, 11, 13]. These traits make *Microsporidia MB* a promising target for blocking malaria transmission [14]. Currently, there is no technique available to culture *Microsporidia MB* spores. Therefore, releasing *Microsporidia MB*-infected mosquitoes that have vertically inherited *Microsporidia MB* from their mothers through mass-rearing in the laboratory, is currently the most likely application scenario to spread *Microsporidia MB* in wild mosquito populations. Although mosquito releases have been controversial due to various issues including community concerns in the past, recent efforts in Africa, supported by increased community engagement, have made them possible [15–20]. This has resulted in the successful release of genetically modified mosquitoes in Burkina Faso and Djibouti [16, 21–23]. In a recent study, 81% of the household heads interviewed expressed a willingness to accept, and 96% were willing to participate in a *Microsporidia MB*-based strategy involving mosquito release [24].

The success of *Microsporidia MB*-infected mosquito release relies on the rate at which *Microsporida MB* is transmitted through the wild mosquito populations. High vertical transmission rates have been recorded in the laboratory from field-collected, blood-fed mosquitoes to the offspring [9–11]. The average vertical transmission from infected field-collected *Anopheles arabiensis* females was reported to be approximately 97% [25]. Similarly, field-collected *Anopheles gambiae *sensu stricto (*s.s.*) females transmitted *Microsporidia MB* infection to their progeny at rates ranging from 0 to 100% across 12 isofemale lines, and from 28.6% to 85.3% in pooled lines [11]. To date, mating remains the only known method for horizontal transmission of *Microsporidia MB* in mosquitoes, with generally higher transmission rates reported from infected males to uninfected females than from infected females to uninfected males [13].

Previous studies using virgin *An. gambiae s.s*. males that were infected with *Microsporidia MB* to study experimental transmission of symbiont demonstrated successful transmission to at least one female in 36% of cages (4 out of 11) [11]. In contrast, infected *An. gambiae s.s.* females only transmitted the infection to at least one male in 11% of cages (1 out of 9) [11]. A similar pattern of lower female-to-male transmission was noted in *An. arabiensis*, where transmission was confirmed in 56% of cages (9 out of 16) with infected males and uninfected females, compared to 33% (5 out of 15) of cages with infected females and uninfected males [13]. These observed modes of vertical and horizontal transmissions are supported by the localization of *Microsporidia MB* in the testis and ovaries [13, 26]. *Microsporidia MB*, when present, appears to be the predominant reproductive eukaryotic microbe in *An. arabiensis* owing to its occurrence in high densities in the gonads as shown by light microscopy [26, 27]. However, it has been shown that the density of *Microsporidia MB* in the gonads and ovaries significantly decreased with mosquito age, with females losing *Microsporidia MB* already between the age of 2–14 days, while in males the decrease was only observed between 7 and 14 days [26]. The effect of male and female mosquito age on horizontal transmission of *Microsporidia MB* is not well understood at present.

*Anopheles* female mosquitoes tend to mate once and at an early stage of their adult life, while male mosquitoes can mate more than once [28]. Moderate to large body size of males is associated with higher mating success [29–31]. Similarly, the bigger body size of females is also associated with a higher probability of being inseminated [32]. However, parasites and symbionts can influence the mating success of the infected host. In the case of parasites, many species of females avoid mating with males with transmittable parasites to prevent acquiring infections and to ensure that the future progeny is not affected by the negative physiological effects of a parasite-infected parent [33–36]. In the case of symbionts, it is expected that the females will prefer males that are infected with symbionts. However, the evidence of female preference for symbiont-infected males is scanty. Studies carried out with *Wolbachia*, a symbiotic bacteria, showed that males infected by the benign wMel *Wolbachia* strain, or the virulent wMelPop (popcorn) strain were equally competitive in mating with females like the uninfected males [31]. However, *Wolbachia* and other symbionts, such as *Rickettsia*, *Spiroplasma,* spread through mechanisms that affect host sex ratio, sex allocation, or sexuality [37]. *Microsporidia MB*, on the other hand, has no negative fitness effects on the sex and does not distort the sex ratio [9].

In the initial stages following the release of *Microsporidia MB*-infected mosquitoes*,* the spread of *Microsporidia MB* infection in the wild mosquito populations will rely on the success of mating between the released laboratory colonies and the wild mosquitoes. Therefore, in this study, the aim was to understand the factors that could influence *Microsporidia MB* transmission and whether *Microspridia MB* infection impacts male mating frequency and competitiveness. Specifically, the; (i) effect of male and female age on *Microsporidia MB* transmission, (ii) effect of *Microsporidia MB* infection on male mating frequency and competitiveness, and (iii) prevalence of *Microsporidia MB* infected males in natural swarms. The key findings of this study will offer valuable insights to inform future research, including the theoretical models used to design a *Microsporidia MB*-based application strategy. Additionally, they will enhance understanding of strictly symbiont-mediated sexual selection.

## Methods

### Mosquito collection and processing

Indoor resting gravid *Anopheles* female mosquitoes were collected using a manual aspirator from houses near the Ahero irrigation scheme (− 34.9190 W,− 0.1661 N), Kisumu County. At this site, *An. gambiae *sensu lato (*s.l.*) is reported to be the most abundant [38], with > 97% of the complex members being identified as *An. arabiensis*. The mosquito collections were kept in cages (30 cm × 30 cm × 30 cm) for transportation to the insectary at the International Centre for Insect Physiology and Ecology-Thomas Odhiambo Campus (*iTOC*-Mbita, Homa Bay County). To minimize mosquito mortality, the cages were covered with wet towels to maintain high humidity levels and mosquitoes had access to 6% glucose. At the insectary, each gravid female mosquito was placed in a 1.5 ml microcentrifuge tube, containing a strip of wet Whatman paper, to force oviposition [39]. The mosquitoes that oviposited were morphologically identified.

### DNA extraction and molecular identification of mosquitoes

Total DNA was isolated from mosquito samples and purified using the protein precipitate method (Puregene, Qiagen, Netherlands) [9]. DNA samples were stored at − 20 °C for short-term storage and − 80 °C for long-term storage. A molecular assay based on the SINE S200 × 6.1 locus was used to differentiate between *An. gambiae s.s.* and *An. arabiensis* [40]. All experiments were carried out using F1 offspring from females who were identified as *An. arabiensis*.

### Molecular detection and quantification of *microsporidia MB* in mosquitoes

Field-collected female mosquitoes identified morphologically as *An. gambiae s.l.* were further identified using molecular techniques as *An. arabiensis* through molecular identification and mosquitoes harvested after experiments were screened for *Microsporidia MB* with qPCR. A 10 μl final PCR reaction volume was used consisting of 2 µl HOTFirepol® Blend Master Mix Ready-To-Load (Solis Biodyne, Estonia, mix composition: 7.5 mM magnesium chloride, 2 mM of each dNTPs, HOT FIREPol® DNA polymerase), 0.5 µl of 5 pmol µl^−1^ of both forward and reverse primers (MB18SF: CGCCGGCCGTGAAAAATTTA and MB18SR: CCTTGGACGTGGGAGCTATC), 2 µl of the template and 5 µl nuclease-free PCR water. PCR thermocycling conditions were as follows: initial denaturation at 95 °C for 15 min, denaturation at 95 °C for 1 min, followed by annealing at 62 °C for 90 s and extension at 72 °C for a further 60 s, all done for 35 cycles. Final elongation was done at 72 °C for 5 min. The scores and densities were normalized against the *Anopheles* ribosomal S7 host gene S7 F: TCCTGGAGCTGGAGATGAAC and S7R: GACGGGTCTGTACCTTCTGG) [41]. Samples were considered negative if the cycle threshold was greater than 30 and if the melt curve did not align with the positive control melt curve. All reactions were done using a Mic qPCR cycler (Biomolecular Systems, Australia).

### Rearing F1 mosquito progeny

All larval offsprings of *Microsporidia MB*-infected female mosquitoes were reared together to establish an infected line. Similarly, a *Microsporidia MB* negative isogroup line was also established. The larvae were reared under ambient semi-field conditions and fed on Tetramin™ baby fish food daily. The pupae that developed from the larvae were sexed as males and females based on their genital lobes under a microscope and then transferred to holding cages, designated for males or females (15 cm × 15 cm × 15 cm), in small cups with water. The adult mosquitoes that emerged from the pupae in the holding cages were maintained on 6% glucose. These adults, F1 progeny of *Microsporidia MB* positive and negative females, and insectary *An. arabiensis* mosquitoes obtained from the insectary at the International Centre for Insect Physiology and Ecology (*icipe*)-Thomas Odhiambo Campus were used for the experiments. As vertical transmission from mother to offspring is not 100% [9], the F1 from *Microsporidia MB* positive line were screened for *Microsporidia MB* presence after the experiment ended to determine if they were positive or negative for *Microsporidia MB*.

### Wing length measurement

The body size of male and female mosquitoes is associated with successful swarming and reproductive success [29–32]. To adjust for any difference in body size of the F1 mosquitoes, wing length used as a proxy for body size, was measured for all the mosquitoes [42, 43]. Before measurements, the mosquitoes were immobilized on ice and then the right wing was carefully detached from the thorax. The ventral side of the wing was measured in millimeters from the notch of the alula to the wing tip using a Dino-lite handheld digital microscope (Model AM4113ZT, Huatang Optical Industry Co., Taiwan).

### Dissection to determine mating status in female mosquitoes

To determine if the experimental female mosquitoes had mated, their spermatheca was dissected. Each female was placed on a slide under a microscope (MOTIC SMZ- 140-N2GG model) and a drop of 1 × Phosphate Buffer Solution (1 × PBS) was added. The microscope was focused until a clear image of the mosquito was observed. Dissecting needles were used to nip the second abdominal segment from the bottom on both sides and then carefully ripped apart from the rest of the body. The presence or absence of sperms in each spermatheca was recorded. A spermathecae with sperms appeared translucent and indicated that the female mosquito had mated and a spermatheca without sperms appeared “transparent” and indicated that the female mosquito had not mated [44].

### The effect of age on horizontal transmission of *Microsporidia MB* in *An. arabiensis*

To determine the effect of age on *Microsporidia MB* transmission, virgin males and females were crossed from either of the three age groups: 3–4 days, 7–8 days, and 10–11 days. Control crosses consisted of males and females *An. arabiensis* mosquitoes from the insectary. Experimental crosses consisted of F1 males (mates) from *Microsporidia MB* infected line with insectary females (recipients) for male-to-female transmission and F1 females (mates) from *Microsporidia MB* infected line with insectary males (recipients) for female-to-male transmission (Table 1). Each cross was replicated at least three times. The sample size per replicate ranged between 18–40 mosquitoes due to the availability of F1 mosquitoes. However, each replicate consisted of males and females combined in an approximately 1:1 ratio. The male and female mosquitoes were combined for 3 days during which they had access to a 6% glucose solution. After this period the mosquitoes were harvested, the wing length of all the mosquitoes was measured, female mosquitoes were dissected to determine their mating status, and all the mosquitoes in the experimental crosses were screened for *Microsporidia MB*, as described above.

**Table 1:** Overview of crosses to determine the effect of age on horizontal transmission of *Microsporidia MB* in *An. arabiensis* mosquitoes. *Microsporidia MB*-negative insectary mosquitoes (recipients) were crossed with *Microsporidia MB-*positive F1 mosquitoes (mates)

Age group	Male		Female	
	Insectary/F1	*Microsporidia MB*	Insectary/F1	*Microsporidia MB*
Control crosses				
3–4 days	Insectary	Uninfected	Insectary	Uninfected
7–8 days	Insectary	Uninfected	Insectary	Uninfected
10–11 days	Insectary	Uninfected	Insectary	Uninfected
Experimental crosses				
Male-to-female transmission				
3–4 days	F1	Infected	Insectary	Uninfected
7–8 days	F1	Infected	Insectary	Uninfected
10–11 days	F1	Infected	Insectary	Uninfected
Female-to-male transmission				
3–4 days	Insectary	Uninfected	F1	Infected
7–8 days	Insectary	Uninfected	F1	Infected
10–11 days	Insectary	Uninfected	F1	Infected

### Comparing the mating frequency of *Microsporidia MB* infected and uninfected males

Male *Anopheles* mosquitoes can mate more than one time; therefore the experiment was designed to enable measuring the mating frequency of each male mosquito. One 3–4 days old virgin male mosquito, either *Microsporidia MB*-infected or *Microsporidia MB*-uninfected F1 male, was placed together with three 3–4 days old virgin insectary female mosquitoes in a paper cup for three days. After three days, the females were removed and another batch of three 3–4 days old virgin females was added. This was repeated until the male mosquito died. All mosquitoes had ad libitum access to 6% glucose solution. The experiment was replicated with male mosquitoes that were the progeny of females collected at different times. Wing length was measured for all the dead males. The females were dissected to determine if the female had mated or not. All mosquitoes were screened for the molecular detection and quantification of *Microsporidia MB.*

### Comparing the mating competitiveness of *Microsporidia MB*-infected and uninfected males

To compare the mating competitiveness of *Microsporidia MB* infected and uninfected F1 males directly, male mosquitoes were combined in the same cage with the uninfected female insectary mosquitoes. A fluorescent dye Rhodamine B was used to differentiate between *Microsporidia MB*-infected and uninfected males [45]. Preliminary experiments were conducted to determine the optimal concentration of the dye that would be detectable in the male for over 7 days and transfer from males to the females on mating. The 0.2% Rhodamine B dye (w/v) in 6% glucose was found to be the optimal concentration. To adjust for any potential effect of Rhodamine B on mating competitiveness, two sets of experiments were conducted: (a) 10 *Microsporidia MB* infected males were fed on Rhodamine B and combined with 10 uninfected males and 20 uninfected females, (b) 10 *Microsporidia MB* uninfected males were fed on Rhodamine B and combined with 10 infected males and 20 uninfected females. The mosquitoes were maintained on 6% glucose and harvested after 3 days. The wing length of the harvested mosquitoes was measured. The females were dissected to determine their mating status and presence of dye in their spermatheca under the microscope (Carl Zeiss Suzhou Co., Ltd., Suzhou, China mounted with NightSEA green filters (SFA-LFS-GR) (Electron Microscopy Sciences, Hatfield, PA). Undissected male mosquitoes were observed under a microscope for Rhodamine B dye absence or presence and screened to confirm the *Microsporidia MB* infection. The experiment was replicated five times each with Rhodamine B-fed *Microsporidia MB*-infected mosquitoes and Rhodamine B-fed uninfected mosquitoes.

### Comparing the proportion of *Microsporidia MB*-infected males in swarming mosquitoes and those caught outside swarms

The collection of male mosquitoes from swarms and outside swarms were done in Ahero, near the Ahero irrigation scheme (− 34.9190 W, − 0.1661 N), Kisumu County. A preliminary survey was conducted to identify the swarming sites and timings. The collection was done daily for two weeks in July 2023. The swarming started at approximately 1840 h and lasted for approximately 30 min. Once the swarming started, a sweep net (15 cm in diameter and 30 cm deep with a wooden handle that was adjusted according to the swarm height) was used to catch the swarming mosquitoes by sweeping multiple times through the swarm until the swarming stopped, or until it was too dark to see the swarm. The mosquitoes collected were knocked out using ethyl acetate and the mosquitoes were separated as males and females and placed individually into labelled 1.5 ml Eppendorf tubes. In the laboratory, the wing length of each mosquito was measured, and all female mosquitoes were dissected to check for the absence or presence of sperms in their spermathecae. All mosquitoes identified morphologically as *An. gambiae* s.l. were identified to species level with PCR and screened to detect and quantify *Microsporidia MB* at the *icipe*-Thomas Odhiambo campus (Homa Bay County). Eight CDC Miniature Light Traps (model 512) were used for indoor and outdoor collection of males outside swarms. Trapping was done in eight houses; four houses had the traps hung outdoors from the eaves and four had traps hung indoors. The traps were set for a 12 h duration, from 1800 tO 0600 h every day for two weeks. The collections were done in locations near and on the same days as the swarm collection. The mosquitoes were then morphologically identified, and *An. gambiae s.l.* males were placed individually in labeled Eppendorf tubes. Following wing length measurement, the males were screened to detect and quantify *Microsporidia MB* and identify mosquito species at the *icipe*- Thomas Odhiambo campus.

### Data analysis

All statistical tests were performed on version 8.0c software and R (version 4.3.2). The replicates of the age group and mating competitiveness experiment that were found to have no *Microsporidia MB-*infected F1 male or female mosquitoes after screening were not included in the analysis. Similarly, replicates of the mating frequency experiments where F1 male mosquitoes were *Microsporidia MB*-uninfected were also not included in the data analysis. Wing lengths were compared with ANOVA. Age groups were compared separately and combined to estimate the overall transmission rate. The transmission rate was a measure of the ability of *Microsporidia MB* to transmit itself and not the absolute transmission. The transmission rate was considered a 100% if every mating with an infected donor leads to *Microsporidia MB* transmission to the recipient (Additional file 1 Age group). The transmission rate (%) was calculated as follows:

$$Observed \,\,transmission \,\,rate \left(\%\right)=\frac{O}{E} \times 100$$

where O is the number of recipients that acquired the infection through mating and E is the expected number of acquired infections if the transmission rate is 100% calculated as a product of the proportion of F1 mates/females that were *Microsporidia MB*-infected and the confirmed number of matings per cage. The mating rate of female mosquitoes was calculated as:

$$Female \,mating \,rate \left(\%\right)=\frac{The \,number \,of \,females \,confirmed \,mated}{Total \,number \,of \,females \,in \,the \,cage}\times 100$$

Linear regression analysis was used to determine if there is a correlation between: i) the *Microsporidia MB* density in the recipient mosquito (predictor variable) and the *Microsporidia MB* density in the potential donor mate (response variable), ii) the *Microsporidia MB* density of the donor mates in crosses where transmission occurred (predictor variable) and *Microsporidia MB* density in donor mates where transmission did not occur (response variable), and iii) *Microsporidia MB* density of the male (predictor variable) and the number of times the male mosquito mated (response variable), in the mating frequency experiment.

The number of times *Microsporidia MB*-infected or *Microsporidia MB*-uninfected males mated from when they were 3–4 days old to death was compared with a t-test. The proportion of male mosquitoes that mated different number of times, were compared with the Chi-square test. Mating competitiveness was, also, compared with the Chi-square test with the proportion of females that mated with *Microsporidia MB*-infected or uninfected males. Wing lengths were compared with a t-test if there were two groups and ANOVA if there were more than two (2) groups. The proportions of *Microsporidia MB*-infected male mosquitoes caught from swarms and outside swarms were compared with the Chi-square test. Statistical significance level was set at α = 0.05.

## Results

### Horizontal transmission of *Microsporidia MB* occurs across age groups

**Fig. 1:**
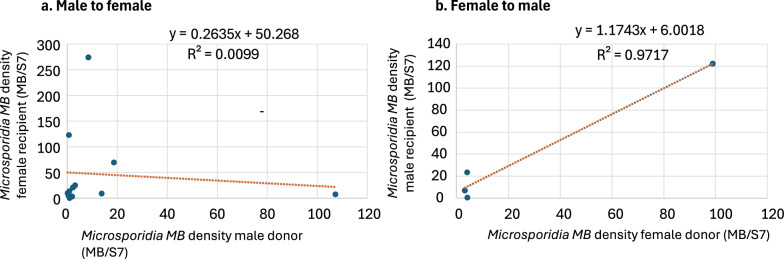
Correlation between the *Microsporidia MB* density in the donor and the recipient, combined for all age group in **a** male to female and **b** female to male transmissions

*Microsporidia MB* was horizontally transmitted from male to female and from female to male (Table 2). The observed transmission was heterogenous and although the trend indicates a higher overall transmission (%) from male to female (41.5% ± 16.9) compared to female to male (22.4% ± 12.4), it was insignificant (χ^2^ = 9.1, df = 9, *p* = 0.42) (Additional file 1_Age group). The *Microsporidia MB* density in the donor mosquitoes was not different in males (R = 0.1, F = 0.2, *p* = 0.6) or females (R = 0.2, F = 0.3, *p* = 0.6) in experiments where transmission occurred or did not occur. The observed transmission rate from males to females was similar across all age groups; there was no significant difference in transmission between mosquitoes of ages 3–4 days old, 7–8 days old, and 10–11 days old (χ^2^ = 11.6, df = 12, *p* = 0.47). Similarly, the observed transmission rate was similar across two age groups for female-to-male crosses; there was no significant difference in transmission between mosquitoes of ages 7–8 days old, and 10–11 days old (χ^2^ = 2.9, df = 3, *p* = 0.41). However, no transmission was observed from 3 to 4 days-old females to males. The mating rate or mosquito body size did not affect the reported transmission rates as there was no difference in male wing length (F = 0.7, df = 2, *p* = 0.5) and female wing length (F = 1.65, df = 2, p = 0.2) or female mating rate (χ^2^ = 14, df = 14, *p* = 0.43) across age groups (Table 2). Due to few transmissions, data was combined across age groups to determine the correlation between the *Microsporidia MB* density in the donor and recipient mosquitoes. In male-to-female crosses, no correlation was found between the *Microsporidia MB* density in the potential donor male and the recipient female (R = 0.1, F = 0.1, *p* = 0.7). However, in female-to-male crosses, the higher *Microsporidia MB* density in potential female donors was positively associated with higher *Microsporidia MB* density in recipient males (R = 0.98, F = 68.5, *p* = 0.01) (Fig. 1).

**Table 2:** Average mating rate (%), average wing length (S.D.), and observed *Microsporidia MB* transmission rate (%) in mosquito mating pairs (*Microsporidia MB*-infected F1 and uninfected insectary) in different age groups

Age group (days)	Male	Female	No. of replicates	Average mating rate (%)	Average wing length S.D		Average transmission rate (%) (S.E.)
					Female	Male	
Control crosses							
3–4	Insectary	Insectary	6	40 (8.7)	3.04 (1.11)	2.84 (0.14)	0
7–8	Insectary	Insectary	7	37.7 (6.3)	3.22 (0.09)	2.98 (0.20)	0
10–11	Insectary	Insectary	6	31.4 (5.2)	3.06 (0.20)	2.92 (0.21)	0
Experimental crosses							
Male-to-female transmission							
3–4	F1	Insectary	4	27.7 (11.3)	3.16 (0.41)	2.98 (0.09)	47 (47)
7–8	F1	Insectary	10	40.4 (7.3)	3.24 (0.29)	3.03 (0.29)	25 (16)
10–11	F1	Insectary	6	32.7 (3.4)	3.02 (0.38)	2.95 (0.30)	65.6 (42.5)
Female-to-male transmission							
3–4	Insectary	F1	3	34.5 (7.8)	3.27 (0.09)	2.89 (0.30)	0
7–8	Insectary	F1	10	31.6 (5.6)	3.34 (0.17)	3.01 (0.22)	31 (21)
10–11	Insectary	F1	6	39.4 (4.4)	3.00 (0.27)	2.88 (0.27)	19 (19)

### Mating frequency of *Microsporidia MB*-infected and uninfected males

**Fig. 2:**
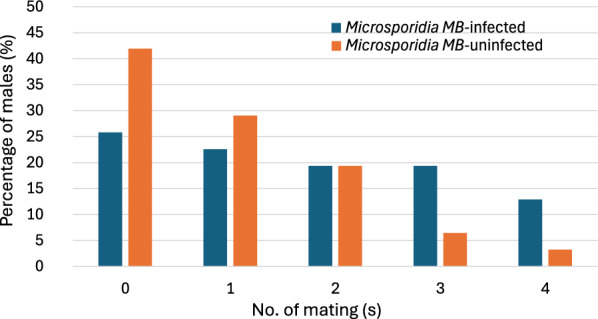
The percentage of *Microsporidia MB*-infected males (n = 31) and *Microsporidia MB*-uninfected males (n = 31) mating different numbers of times between the age of 3–4 days old to death

*Microsporidia MB*-infected males mated almost twice as much as *Microsporidia MB*-uninfected F1 males. On average *Microsporidia MB*-infected males mated 1.7 (S.E. = 0.25) times and *Microsporidia MB*-uninfected F1 males mated 1 (S.E. = 0.19) time between the age of 3–4 days old and death (t = 2.2, df = 56.8, p = 0.03). A significantly higher proportion of *Microsporidia MB*-infected males mated 3–4 times compared to *Microsporidia MB*-uninfected males (χ^2^ = 4.69, df = 1, *p* = 0.03) (Fig. 2). Only one *Microsporidia MB*-infected male mated in round 6 (estimated age during the 6 th round, 21–24 days) of the experiment. *Microsporidia MB*-uninfected males mated only up to round 5 (estimated age during the 5 th round, 18–21 days) (Additional file 2_Mating rate). No correlation was found between the *Microsporidia MB* density and the number of times *Microsporidia MB*-infected males mated (R = 0.1, F = 0.8, *p* = 0.4).

### Mating competitiveness of *Microsporidia MB*-infected and uninfected male mosquitoes

**Fig. 3:**
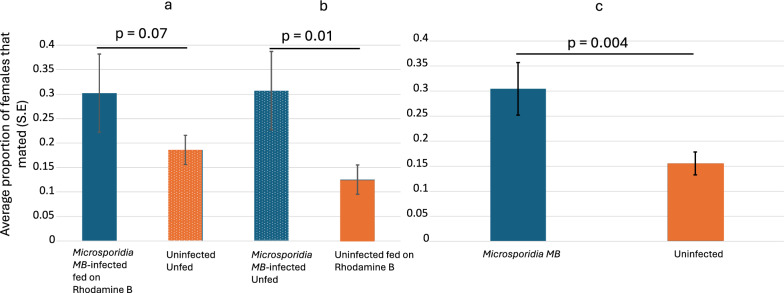
The average proportion of females (S.E) that mated with *Microsporidia MB* infected versus uninfected males **a** Rhodamine B fed to *Microsporidia MB*-infected male mosquitoes **b** Rhodamine B fed to uninfected male mosquitoes and **c** Combined Rhodamine fed and unfed *Microsporidia MB*-infected and uninfected male mosquitoes

**Fig. 4:**
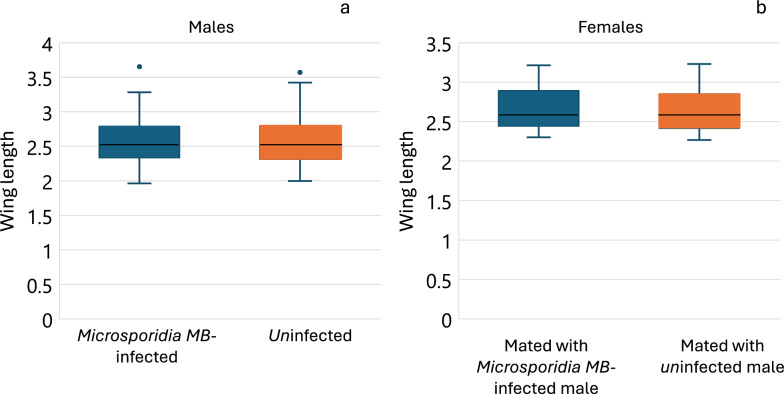
Box plot of wing length (in mm) of **a** *Microsporidia MB*-infected and uninfected males and **b** wing length of females that mated with *Microsporidia MB*-infected males and uninfected males

Almost twice the females (Average proportion: 0.3 vs 0.13) mated with *Microsporidia MB*-infected male mosquitoes fed on Rhodamine B than uninfected males (χ^2^ = 3.13, df = 1, *p* = 0.07), but this was not significant (Fig. 3a; Additional file 3 Mating competitiveness). Higher mating competitiveness was also observed for *Microsporidia MB*-infected when uninfected mosquitoes were fed on Rhodamine B instead of *Microsporidia MB*-infected (Fig. 3b) (χ^2^ = 5.76, df = 1, *p* = 0.01). Comparing *Microsporidia MB*-infected and uninfected male mosquitoes, irrespective of which group was fed on Rhodamine B also showed twice the mating competitiveness (χ^2^ = 8.45, df = 1, *p* = 0.004) of *Microsporidia MB*-infected male mosquitoes (Fig. 3c). The wing lengths of *Microsporidia MB*-infected and uninfected male mosquitoes were not significantly different (t = − 0.6, df = 140, *p* = 0.5). Similarly, the wing lengths of females that mated with the *Microsporidia MB*-infected and uninfected male mosquitoes were also not significantly different (t = 0.002, df = 45, *p* = 0.9) (Fig. 4).

### Males* Microsporidia MB* prevalence in swarms and outside swarms

In total, 653 *An. arabiensis* males were caught indoors and outdoors outside swarms, and 3194 swarming *An. arabiensis* males were caught from a total of 30 swarms. In the swarm collection, 87 (2.7%) of the male mosquitoes were infected with *Microsporidia MB* which was significantly higher than the 6 (0.9%) in the male mosquitoes (χ^2^ = 7.514, df = 1, *p* < 0.0061) caught outside swarms. The average wing length of swarming males was 2.39 (S.D., 0.43), and of males caught outside swarms was 2.43 (S.D., 0.72); there was no significant difference between the wing lengths of two groups (t = − 0.9, df = 439, *p* = 0.3).

## Discussion

In this study a moderate, although heterogenous, horizontal transmission of *Microsporidia MB* was recorded especially from male mosquitoes to female mosquitoes. *Microsporidia MB*-infected male mosquitoes also showed a higher mating frequency and competitiveness compared to uninfected males in the laboratory. The prevalence of *Microsporidia MB*-infected males was also found to be higher in swarms compared to the dispersed male population at the same field site.

Transmission heterogeneity in host-parasite systems is not uncommon and can be due to environmental, physiological, behavioural, and genetic factors [46, 47]. Mating success is also known to be very variable [48]. In this study, mating rate, and mosquito body size do not explain the heterogeneity. *Microsporidia MB* density in the donor male or female mosquitoes also does not account for the heterogeneity. Mosquitoes were collected from the same field site and any significant variation in the *Microsporidia MB* strain is unlikely. The mosquito collection and experiments were, however, carried out over time during which, the climatic conditions may have affected the horizontal transmission rate [9, 13]. Identifying and testing possible factors that cause heterogeneity will be essential in developing a super-spreading strategy for* Microsporidia MB.*

Similar to previous studies the horizontal transmission rate of *Microsporidia MB* was higher from male to female compared to female to male [11, 13]. Previously it was shown that *Microsporidia MB* infective spores were transferred with the ejaculation to the female resulting in higher transmission [13]. In the case of female-to-male, the mode of transfer is being investigated, but it may be because little to no tissue or fluid is transferred from female to male during copulation.

In this study, no *Microsporidia MB* transmission was reported from 3–4 day old *An. arabiensis* females to males. In *An. gambiae s.s.,* the horizontal transmission was observed from female to male but in 5–7 days mosquitoes [11]. The study on horizontal transmission in *An. arabiensis* did not specify the age of the adults and, therefore, it is not possible to compare [13]. *Microsporidia MB* is already present in the guts and gonads of 2-day-old female and male mosquitoes and there is a decline in *Microsporidia MB* density between days 7 and 14 [26]. This indicates that the density of *Microsporidia MB* is not the only factor important for horizontal transmission, as male mosquitoes transmitted *Microsporidia MB* in all three age groups tested, ranging from 3 to 14 days. *Microsporidia MB* male mosquitoes mated at an estimated 21–24 day old male mosquitoes in the mating competitiveness experiment. In nature, female mosquitoes mate once and earlier in their lifetime during which no horizontal transmission was observed. Male mosquitoes on the other hand mate multiple times and are more efficient in transmitting *Microsporidia MB*. This supports the male-only mosquito release approach which is expected to have a higher acceptance in the community due to fear of an increase in mosquito bites if mosquitoes are released in the proximity of the homesteads [24].

*Microsporidia MB* density in the infected males was not related to the *Microsporidia MB* density in the recipient females, successful transmission, or the number of times the male mosquito mated. Previous studies also showed that the *Microsporidia MB* density of the male mosquitoes is not related to horizontal transmission or the number of matings and suggested that this may be because the density represents the *Microsporidia MB* localized not only in the gonads but also in the midgut and fatty bodies; correlation may be found if only gonad *Microsporidia MB*-density is considered [13, 26]. Interestingly, this study reports that *Microsporidia MB* density in the infected female was related to *Microsporidia MB* density in the recipient males. In *Anopheles* mosquitoes the ovaries are considerably larger than the testis; the testis is approximately 0.4 mm in length while the ovary is 2 mm in length [49, 50]. It may be that the female *Microsporidia MB* density in the ovaries is more representative of the total body load. In the females, there is a proliferation of *Microsporidia MB* following a blood meal, however, this was not the case in this study as the females were not provided a blood meal [26]. Also noteworthy is that the number of transmission events from female to male mosquitoes was few and more data is required to establish the correlation.

From 3–4 days to death, the mating frequency of *Microsporidia MB-*infected male mosquitoes was found to be almost double that of uninfected male mosquitoes. Also, more females mated with *Microsporidia MB*-infected male mosquitoes compared to uninfected males. This partially explains the higher-than-expected transmission rate in the age group experiments. Male mosquitoes need to successfully mate with wild females for strategies based on reproductive control such as sterile insect technique, transgenic mosquitoes, and *Wolbachia* to be successful [48]. Generally, if the mating competitiveness of the male mosquitoes is equivalent to their wild competitors or even slightly lower, it is considered sufficient [30, 51–53]. These results suggest that a strategy based on releasing male *Microsporidia MB-*infected mosquitoes to increase *Microsporidia MB* prevalence in natural populations is likely to be successful. This is also supported by data that shows that a higher proportion of males caught from swarms were *Microsporidia MB*-infected compared to those outside swarms.

This study was conducted with F1 progeny of field-collected *Microsporidia MB*-infected *An. arabiensis* mothers and are, therefore, representative of the natural population. However, it is noticeable that insectary mosquitoes used in the experiments were comparable in mating rate and wing size, which may be due to the semi-field rearing conditions. Mosquito releases will require colonization and mass production of *Microsporidia MB*-infected mosquitoes which can influence the mating competitiveness [48, 54, 55]. Mass production under semi-field conditions may assist in the preservation of mating competitiveness allowing the released mosquitoes to disperse over considerable ranges, locate appropriate mating venues, and compete with wild males but will also introduce the risk of seasonal population crashes and variation in the quality of mosquitoes produced [56].

This study offers promising insights into the horizontal transmission of *Microsporidia MB* in *Anopheles* mosquitoes. However, the mechanisms underlying the increased mating competitiveness of *Microsporidia MB*-infected male mosquitoes require further investigation. Symbionts may influence host odour, potentially making them more attractive, or enhancing the host's overall development and reproductive fitness [57, 58]. Also, transmission to females in subsequent matings and mating preference of *Microsporidia MB-*infected males for *Microsporidia MB-*infected versus uninfected females need to be established. Future research must also determine if the higher mating frequency and competitiveness translate to more offspring. The results will also need to be validated in semi-field conditions.

In conclusion, *Microsporidia MB* is transmitted horizontally through mating, with *Microsporidia MB*-infected male mosquitoes demonstrating nearly twice the competitiveness of their uninfected counterparts. These findings provide valuable insights for developing strategies to disseminate *Microsporidia MB* in natural mosquito populations for malaria control, particularly through male mosquito releases. However, further research is needed to understand the mechanisms driving increased mating frequency and competitiveness and to validate these findings under semi-field conditions.

## Supplementary Information

Additional file 1

Additional file 2

Additional file 3

## Data Availability

Data is provided within the manuscript or supplementary information files.
